# Sedative and hypnotic effects of compound Anshen essential oil inhalation for insomnia

**DOI:** 10.1186/s12906-019-2732-0

**Published:** 2019-11-11

**Authors:** Yu Zhong, Qin Zheng, Pengyi Hu, Xiaoying Huang, Ming Yang, Guilin Ren, Qing Du, Jun Luo, Kenan Zhang, Jing Li, Haixia Wu, Yuanyuan Guo, Shanshan Liu

**Affiliations:** 1 Key Laboratory of Modern Preparation of Traditional Chinese Medicine, Ministry of Education, State Key Lab.of Innovation Drug and Effcient Energy-Saving Pharmaceutical Equipment, Jiangxi University of Traditional Chinese Medicine, Nanchang, 330004 China; 2 Traditional Chinese Medicine hospital Affliated to Southwest Medical University, Luzhou, 646000 China

**Keywords:** Insomnia, Aromatherapy, Compound Anshen essential oil, Sedative and hypnotic, Inhalation

## Abstract

**Backgrounds:**

The chemical composition of many essential oils indicates that they have sedative and hypnotic effects, but there is still a lack of systematic studies on the sedative and hypnotic effects of essential oils. In addition, aromatherapy does not seem to have the side effects of many traditional psychotropic substances, which is clearly worthwhile for further clinical and scientific research. The clinical application of essential oils in aromatherapy has received increasing attention, and detailed studies on the pharmacological activities of inhaled essential oils are increasingly needed.

**Hypothesis/purpose:**

As insomniacs are usually accompanied by symptoms of depression and anxiety of varying degrees, based on the theory of aromatherapy of Traditional Chinese Medicine, this experiment is to study a Compound Anshen essential oil that is compatible with Lavender essential oil, Sweet Orange essential oil, Sandalwood essential oil and other aromatic medicine essential oils with sedative and hypnotic effects, anti-anxiety and anti-depression effects. To study the sedative and hypnotic effects of Compound Anshen essential oil inhaled and the main chemical components of Compound Anshen essential oil, and to compare and analyze the pharmacodynamics of diazepam, a commonly used drug for insomnia.

**Methods:**

The Open field test and Pentobarbital-induced sleep latency and sleep time experiments were used to analyze and compare the sedative and hypnotic effects of inhaling Compound Anshen essential oil and the administration of diazepam on mice. The changes of 5-HT and GABA in mouse brain were analyzed by Elisa. The main volatile constituents of Compound Anshen essential oil were analyzed by gas chromatography-mass spectrometry (GC-MS).

**Results:**

Inhalation of Compound Anshen essential oil can significantly reduce the spontaneous activity of mice, reduce latency of sleeping time and prolong duration of sleeping time. The results of enzyme-linked immunosorbent assay showed that Compound Anshen essential oil can increase the content of 5-HT and GABA in mouse brain. The main volatile chemical constituents of the Compound Anshen essential oil are D-limonene (24.07%), Linalool (21.98%), Linalyl acetate (15.37%), α-Pinene (5.39%), and α-Santalol (4.8%).

**Conclusion:**

The study found that the inhalation of Compound Anshen essential oil has sedative and hypnotic effect. This study provides a theoretical basis for further research and development of the sedative and hypnotic effects of Compound Anshen essential oil based on the theory of aromatherapy.

## Background

Insomnia is a common sleep disorder that can be caused by psychological stress, chronic pain, and medication [1]. A large number of insomnia patients are associated with different degrees of depression and anxiety [2]. These symptoms can occur simultaneously with insomnia, resulting in decreased mental activity, memory loss, slow response, autonomic dysfunction, resulting in decreased immune function and memory loss [3], Insomnia affects approximately 10–30% of adults worldwide [4], and 6% are diagnosed with chronic insomnia [5]. Drugs used to treat insomnia include benzodiazepine receptor agonists, non-benzodiazepine receptor agonists, selective melatonin receptor agonists, and sedative antidepressants [6]. In addition, most of the current targets for anti-insomnia drugs are serotonin (5-HT) receptors [7], γ-aminobutyric acid (GABA) receptors [8]. However, taking these drugs will be accompanied by “hangover” effect, psychomotor disorder, drug dependence, addiction, tolerance, amnesia, rebound insomnia and other side effects, and their clinical efficacy is still controversial [9]. Therefore, the research on new sedative and hypnotic drugs with fewer side effects and better efficacy is still continuing [10].

Aromatherapy is currently used to treat chronic pain, depression, anxiety, insomnia, improve cognitive efficiency, relieve stress and other psychological and physiological conditions related disorders [11]. Aromatherapy is the use of essential oils extracted from the flowers, stems, leaves, roots and fruits of various plants. It is absorbed by the body by oral, inhalation, diffusion, bathing and massage to improve mental and physical health [12]. It has been reported that the main mechanism of aromatherapy may be related to the limbic system of the brain. Aroma components stimulate olfactory cells, which transmit signals to the brain and affect the autonomic nervous system and hormone secretion [13]. Odor particles reach the limbic system through the olfactory nerve, producing sedative and relaxing effects that affect blood pressure, heart rate, memory and stress response [14]. Essential oils act directly on the respiratory, circulatory and central nervous systems through the skin and respiratory tract [15]. The chemical composition of many essential oils indicates that essential oils have a calming and hypnotic effect, but there is still a lack of systematic research on the sedative and hypnotic effects of essential oils. Some studies have shown that certain essential oils have sedative and hypnotic effects, including bergamot, sweet orange, valerian, lemon, rose, cedar and other essential oils [16]. Lavender essential oils mainly include linalool and linalyl acetate. It has been reported that low-dose linalool has a sedative effect on human body by steam inhalation [17]. Previous studies on aromatherapy to improve sleep used inhalation, massage, skin smear and other methods, using lavender, geranium, citrus, bergamot and marjoram and other essential oil mixture, these results also show that aromatherapy to improve sleep is significant [18]. The use of essential oils may be a safe and effective treatment for or in the treatment of insomnia, which may reduce the overuse of prescription drugs and reduce the risk of sleep disorders affecting the health of the body in the short or long term [16]. In addition, aromatherapy does not seem to have the side effects of many traditional psychotropic substances, which is clearly worthwhile for further clinical and scientific research [11]. The clinical application of essential oils in aromatherapy has received more and more attention, and detailed research on the pharmacological activities of inhaled essential oils is increasingly needed.

Inhalation is a fast and effective aromatherapy that induces central nervous system response in just 4 s. It uses respiration to start from the absorption of volatile molecules through the nasal mucosa, while volatile molecules enter the circulatory system after gas exchange into the lungs [19]. The olfactory pathway not only conducts the sense of smell, but also regulates the memory, emotions, visceral activities, and advanced functions of the brain such as alertness and sleep through olfactory regulation. The neurotransmitter of olfactory transmission information and the Orexin neuronal system in the lateral region of the hypothalamus initiate neurons such as brain stem, basal forebrain, and hypothalamus, producing gamma-aminobutyric acid (GABA), Serotonin (5-HT), etc. [20]. These transmitters are closely related to the pathogenesis of insomnia and other diseases. Inhalation administration is a nondestructive way of administration, which minimizes toxic and side effects. Therefore, it may be an effective way to control and treat insomnia and other psychiatric diseases.

In many cases, the role of a single medicine is limited and cannot completely solve the complex and multivariate conditions of insomnia patients. Insomnia is caused by a variety of pathogenic factors. Insomnia patients are usually accompanied by different degrees of depression and anxiety. Therefore, the use of a single essential oil maybe only achieves the effect of sedative and hypnosis, and cannot completely solve the problem of sleep disorders. Multi-herb therapy basing on Traditional Chinese Medicine theory is one of the most important characteristics of Traditional Chinese Medicine clinical practice [21, 22]. Traditional Chinese Medicine believes that insomnia is caused by dysfunction of organs. Clinically, it is more common in patients with Insomnia due to liver depression, which is the main cause of insomnia [23]. Qi-regulating drugs have the effect of dispersing stagnated liver qi to relieve depression, and thus may be an effective medicine for treating insomnia [24]. In Traditional Chinese Medicine, Sweet orange, Sandalwood, Rose, Frankincense, Agarwood, Orange blossom are Qi-regulating drugs [23, 24], which have the effect of dispersing stagnated liver qi to relieve depression. Sweet orange (Orange peel) and Agarwood are classic Herb pairs, which have the effect of relieving qi stagnancy in liver and resolving stagnation for tranquilization. From the perspective of Western medicine, Lavender [25, 26] has sedative-hypnotic effects and anti-anxiety effects, Sandalwood [27] and Agarwood [28] have sedative-hypnotic effects, Sweet orange [29], Rose [30], Frankincense [31] and Orange Blossom all have anti-depression effects. In this study, the Lavender essential oil, a commonly used in the treatment of insomnia, was combined with the Qi-regulating drugs’ essential oil to prepare the Compound Anshen essential oil and its effect on sedative and hypnotic effects with inhalation administration were studied. Based on the theory of aromatherapy of Traditional Chinese Medicine, this experiment is aiming at treating insomnia from the perspective of relieving qi stagnancy in liver and resolving stagnation for tranquilization. The purpose of the study is to investigate the potential treatment on insomnia of Compound Anshen essential oil.

## Methods

### Animals

ICR mice aged 6–8 weeks, half male and half female, weighing 25-35 g, provided by Jiangsu Jicui Yaokang Biotechnology Co., Ltd. License number: SCXK (su) 2018–0008. During the whole experiment, the animals could get enough food and water. The temperature of the feeding environment was 21 °C ± 1 °C, the humidity was 55% ± 5%, the light and dark alternate for 12 h, and the test was started after 1 week of adaptive feeding.

The experiments were approved by the Institutional Animal Ethics Committee of Jiangxi University of Traditional Chinese Medicine. All animals were maintained in accordance with the guidelines outlined by the Chinese legislation on the ethical use and care of laboratory animals. All animals were maintained in accordance with the guidelines outlined by the legislation on the ethical use and care of laboratory animals. All efforts were made to minimize both animal suffering and the number of animals used to produce reliable data.

### Main drugs and reagents

Compound Anshen essential oil is a blended formula consisting of seven natural plant essential oils. Lavender, Sweet Orange, Sandalwood, Frankincense, Orange blossom, Rose, and Agarwood oil are purchased from Puli Aroma Pharmaceutical Technology (Shanghai) Co., Ltd. The seven essential oils are mixed to make a composite oil of a specific mixing ratio. (Lavender, Sweet orange, Sandalwood, Frankincense, Orange blossom, Rose, Agarwood oil blend ratio 10:4:2:1.6:1.2:1:0.6). Diazepam (Beijing Yimin Pharmaceutical Co., LTD). Tween-80 (Shanghai Miura Reagent Co., Ltd). Ethanol Disinfectant (Nanchang Likang Pharmaceutical Machinery Co.,Ltd). 5-HT Elisa kit (Andygene). GABA Elisa kit (Andygene). PBS buffer (Solarbio).

### Main equipment and instruments

Ultrasonic atomizing aromatherapy machine (Shenzhen Kangmeitai Industrial Co., Ltd). Animal aromatherapy room (large box structure of 50x50x40cm made of plexiglass, 4 small boxes structure of 20x20x20cm which can be vented with gas, and ultrasonic atomizing aromatherapy machine can be placed in the middle). SMART Behavior Recording Video Analysis System (Panlab). Agilent 7890A GC-5975 Mass Spectrometer (Agilent, USA). High-throughput tissue grinding machine (Ningbo Xinzhi Biotechnology Co., Ltd. SCIENTZ-192). Low temperature high speed centrifuge (2K15C, SIGMA, Germany). Microplate reader (Gene Co., Ltd. Elx800).

### Establishing mice models of insomnia

Experimental animals except for the control group were given intraperitoneal injection of PCPA to induce animal insomnia. PCPA is an inhibitor of tryptophan hydroxylase (TDH), and TDH is the limit of the sleep neurotransmitter serotonin (5-HT). Speed enzyme, so PCPA deprives sleep by blocking 5-HT. Therefore, PCPA is used to deprive sleep by blocking 5-HT. Each mouse was accurately weighed to PCPA according to the amount of 300 mg/kg, and was mixed with weak alkaline saline to prepare a suspension. After continuous injection for 2 days, after the first intraperitoneal injection for 28–32 h, the circadian rhythm disappeared and the day and night activities continued, indicating successful modeling.

### Grouping and treatment

48 mice were randomly divided into 6 groups. This experiment consisted of control group, model group, diazepam group, and Compound Anshen essential oil groups (low-dose group, medium-dose group, high-dose group). After the establishment of the PCPA insomnia model, the model group did not implement the inhalation of essential oil intervention, in which the low-dose, medium-dose, and high-dose groups were daily aromatherapy for 7 consecutive days, 60 min per day. The essential oil was diluted with 1% Tween 80 Solution. The concentration of low, medium, and high doses of Compound Anshen essential oil was 1 × 10^− 3^, 2 × 10^− 3^, 4 × 10^− 3^. The inhalation time was set at 8:00–16:00 daily. Diazepam was prepared into a solution of 0.1 ml/10 g with distilled water. The diazepam group was given diazepam solution by gavage for 7 consecutive days, and the dose was 1 mg/kg (the dose was converted into the dose of mice by body surface area according to the adult dose). The control group, model group, and each Compound Anshen essential oil groups were given the same volume of distilled water by gavage.

### Weight changes in mice

Mice were weighed before the establishment of the PCPA insomnia model and at the end of the experiment, and the weight gain was calculated.

### Open field test

After the final aromatherapy or drug administration for 30 min, mice in each group were subjected to an open field test. The mice were acclimated to the environment for 5 min, and the behavioral parameters were counted within 3 min, and the differences between the mice in each group were compared. Autonomous activity test index: Total movement distance, unit: cm; Maximum velocity, unit: cm/s; Average velocity, unit: cm/s; Rest time, unit: s; Number of arm lifting.

### Pentobarbital-induced sleeping

Mice in each group were intraperitoneally injected with a threshold dose of pentobarbital sodium of 45 mg/kg after the final aromatherapy or 30 min of administration. After the administration, the righting reflex of the mice disappeared for 1 min as the index of falling into sleep. The time from the injection of the drug to the disappearance of the righting reflex was latency of sleeping time, the time that the righting reflex disappeared until the recovery was duration of sleeping time, and latency of sleeping time and duration of sleeping time were recorded.

### Analysis of brain neurotransmitters

After the open field test was completed, the mice were fasted for 6 h. The mice in each group were sacrificed by cervical dislocation, the skull was removed, brain tissue was exposed, and the brain tissue was completely removed. After separating the brain, the blood and tissues were washed in ice-cold PBS buffer and weighed with an electronic balance. The mouse brain was placed in a centrifuge tube, and a certain amount of PBS buffer was added thereto, thoroughly homogenized, centrifuged (3000 r/min, 20 min), and the supernatant was taken. After treatment with the mouse 5-HT and GABA ELISA kits, the 5-HT and GABA contents were measured at 450 nm on a microplate reader.

### Chemical composition analysis of Compound Anshen essential oil

Gas chromatographic conditions: Agilent DB-624 (30 m×320 μm×1.8 μm) capillary column was used, the carrier gas was high purity He (99.999%), the injection volume was lμL, the split ratio was 40:1, and the flow rate was 1 mL/min. Temperature program: initial temperature 40 °C (for 1 min), ramp to 10 °C/min to 220 °C, and then raise the temperature to 25 °C/min to 280 °C (for 9 min). Mass spectrometry conditions: EI ion source, electron energy 70 eV, ion source temperature 230 °C, MS quadrupole temperature 150 °C;Interface temperature 250 °C, solvent 3.0 min delay, quality scan pattern full scan, scan range of 30 ~ 650 amu. Standard spectral library NIST11 retrieval, peak area normalization method to calculate the relative percentage content of each component.

### Statistical analysis

The test results were expressed as Mean ± SD. SPSS 21.0 software was used for statistical analysis, single factor ANOVA method was used for analysis, LSD method was used for homogeneity of variance, Games-Howell method was used for heterogeneity of variance, and *P* < 0.05 was taken as the test level.

## Results

### Measurement results of body weight changes in mice

**Fig. 1:**
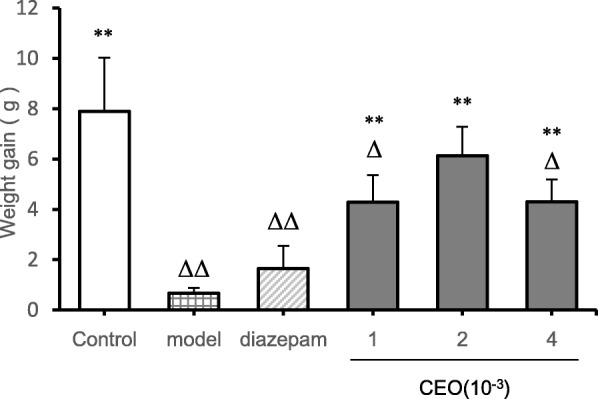
Comparison of weight gain in each group at the end of the experiment ($\overline{x}\pm s$,*n* = 8). The weight gain at the end of the experiment was shown Fig. 1.The low-dose, medium-dose, and high-dose groups were daily aromatherapy, and the daily concentration of low, medium, and high doses of Compound Anshen essential oil (CEO) was 1 × 10^− 3^, 2 × 10^− 3^,4 × 10^− 3^ for 7 consecutive days. Note: compared with control group, ^Δ^*P*<0.05,^ΔΔ^*P*<0.01,compared with model group,^*^P<0.05,^**^P<0.01

Animals were weighed before the establishment of the PCPA insomnia model and at the end of the experiment, and the weight gain was calculated. The effect of Compound Anshen essential oil on animal body weight was studied by weight change experiment and compared with the diazepam group (Fig 1). Compared with the control group, the weight gain of the model group (*P* = 0.000) was extremely significantly reduced, the weight gain of the and the diazepam group (*P* = 0.000) was extremely significantly increased, the weight gain of the low-dose group (*P* = 0.14) and high-dose group (*P* = 0.14) was significantly increased, and the weight gain of the medium-dose group (*P* = 0.370) was not significantly different; Compared with the model group (*P* = 0.237), the weight gain of the other groups (*P* = 0.000) was extremely significantly increased except for the diazepam group.

### Open field test results

**Fig. 2:**
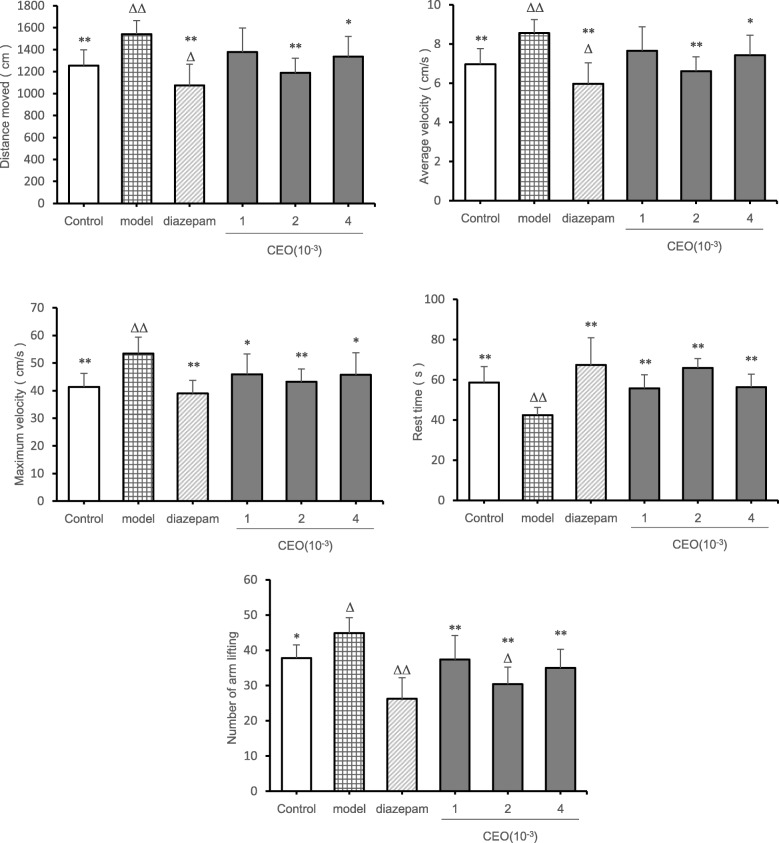
Effect of Compound Anshen essential oil on movement distance, average speed, maximum speed, rest time and number of arm lifting of mice ($\overline{x}\pm s$,n = 8). The experimental results of the autonomic activity of the mice after inhaling the Compound Anshen essential oil are shown in Fig. 2. The low-dose, medium-dose, and high-dose groups were daily aromatherapy, and the daily concentration of low, medium, and high doses of Compound Anshen essential oil (CEO) was 1 × 10^− 3^,2 × 10^− 3^,4 × 10^− 3^ for 7 consecutive days. Note: compared with control group, ^Δ^*P*<0.05,^ΔΔ^*P*<0.01,compared with model group,^*^P<0.05,^**^P<0.01

From the perspective of mice movement distance (Fig. 2), compared with the control group, the movement distance of the model group (*P* = 0.002) was extremely significantly increased, and the movement distance of the diazepam group (*P* = 0.039) was significantly decreased. Compared with the model group, there was no significant difference in the movement distance in the low-dose group (*P* = 0.062), and the movement distance of the control group (*P* = 0.002), the diazepam group (*P* = 0.000) and the medium-dose group (*P* = 0.000) was extremely significantly reduced, and the movement distance of the high-dose group (*P* = 0.020) was significantly increased. From the perspective of average velocity of mice, the average velocity of the model group (*P* = 0.002) increased extremely significantly, and the average velocity of the diazepam group diazepam group (*P* = 0.039) decreased significantly. Compared with the model group, there was no significant difference in the average velocity of the low-dose group (*P* = 0.062), the average velocity of the control group (*P* = 0.002) decreased extremely significantly, the average velocity of the diazepam group (*P* = 0.000) and the medium-dose group (*P* = 0.000) decreased extremely significantly, and the average velocity of the high-dose group (*P* = 0.021) decreased extremely significantly. From the point of view of the maximum velocity of mice, compared with the control group, the maximum velocity of the model group (*P* = 0.000) was extremely significantly increased, and there was no significant difference in the other groups (*P* > 0.05). Compared with the model group, the maximum velocity of the control group (*P* = 0.000), the diazepam group (*P* = 0.000) and the medium-dose group (*P* = 0.002) was extremely decreased extremely significantly, and the maximum velocity of the low-dose group (*P* = 0.017) and the high-dose group (*P* = 0.015) was decreased significantly. From the resting time of the mice, compared with the control group, the model group (*P* = 0.004) had extremely significant difference, which showed an increase in resting time, and there was no significant difference in other groups (*P* > 0.05); Compared with the model group, the other groups had extremely significant differences (*P* < 0.01), which showed an increase in resting time. From the point of view of number of arm lifting of mice, compared with the control group, the model group had significant difference (*P* = 0.010) which showed an increase in the number of arm lifting, while the diazepam group (*P* = 0.000) and the medium-dose group (*P* = 0.008) showed an extremely significant difference, which showed that number of arm lifting decreased. Compared with the model group, there were significant differences in other groups (*P* < 0.05), among which the diazepam group (*P* = 0.000), the low-dose group (*P* = 0.007), the medium-dose group (*P* = 0.000) and the high-dose group (*P* = 0.001) had extremely significant differences, which showed that the number of arm lifting was reduced. From the point of view of number of arm lifting of mice, compared with the control group, the number of arm lifting in the model group (*P* = 0.010) increased significantly, and number of arm lifting in the diazepam group (*P* = 0.000) and the medium-dose group (*P* = 0.008) was extremely significantly reduced. Compared with the model group, the number of arm lifting in the diazepam group (*P* = 0.000), the low-dose group (*P* = 0.007), the medium-dose group (*P* = 0.000), and the high-dose group (*P* = 0.001) was extremely significantly reduced.

### Pentobarbital-induced sleeping

**Fig. 3:**
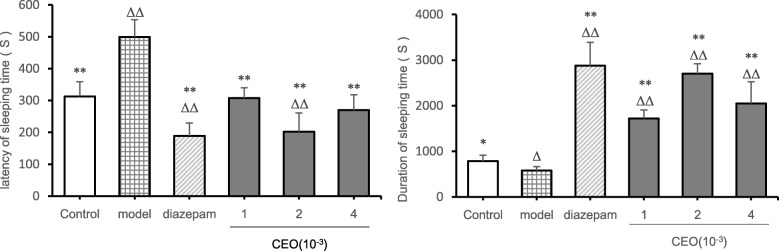
Effect of Compound Anshen essential oil on latency of sleeping time and duration of sleeping time in pentobarbital natrium-induced mice ($\overline{x}\pm s$,n = 8) .The experimental results of latency of sleeping time and duration of sleeping time induced by pentobarbital sodium after inhaling the Compound Anshen essential oil in mice were shown Fig. 3.The low-dose, medium-dose, and high-dose groups were daily aromatherapy, and the daily concentration of low, medium, and high doses of Compound Anshen essential oil (CEO) was 1 × 10^− 3^, 2 × 10^− 3^, 4 × 10^− 3^ for 7 consecutive days. Note: compared with blank group, ^Δ^ *P*<0.05,^ΔΔ^ *P*<0.01,compared with model group,^*^P<0.05,^**^P<0.01

From the perspective of latency of sleeping time (Fig. 3), compared with the control group, the latency of sleeping time of the diazepam group (*P* = 0.000) and the medium-dose group (*P* = 0.000) was extremely significantly shortened, and the latency of sleeping time of the model group (*P* = 0.000) was extremely significantly increased. Compared with the model group, the latency of sleeping time of the other groups was significantly different (*P* < 0.01), which was manifested as decreased latency of sleeping time. From the perspective of duration of sleeping time, compared with the control group, the sleep time of the diazepam group (*P* = 0.000), the low-dose group (*P* = 0.000), the medium-dose group (*P* = 0.000), and the high-dose group (*P* = 0.001) was extremely significantly increased, and the sleep time of the model group (*P* = 0.027) was significantly reduced. Compared with the model group, the duration of sleeping time of the other groups was significantly different (*P* < 0.01), which was manifested as increased duration of sleeping time.

### Analysis of brain neurotransmitters

**Fig. 4:**
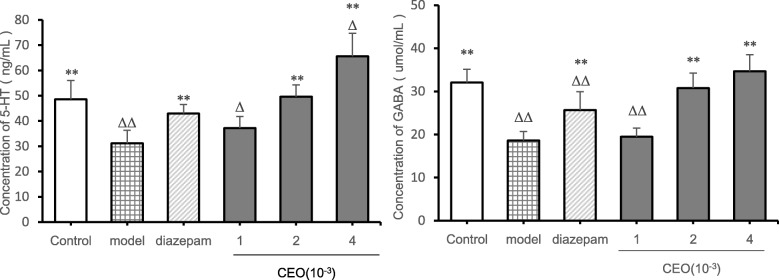
Effects of Compound Anshen essential oil on neurotransmitters in the brain of mice($\overline{x}\pm s$,n = 8). The experimental results of the effects of Compound Anshen essential oil on brain neurotransmitters are shown in Fig. 4. The low-dose, medium-dose, and high-dose groups were daily aromatherapy, and the daily concentration of low, medium, and high doses of Compound Anshen essential oil (CEO) was 1 × 10^− 3^, 2 × 10^− 3^, 4 × 10^− 3^ for 7 consecutive days. Note: The experimental results of the effect of Compound Anshen essential oil on neurotransmitters in the brain are shown in figure 4.compared with control group, ^Δ^ *P*<0.05,^ΔΔ^ *P*<0.01,compared with model group,^*^P<0.05,^**^P<0.01

From the perspective of the concentration of 5-HT in the brain in mice (Fig. 4), compared with the control group, the 5-HT concentration in the model group (*P* = 0.001) was extremely significantly reduced, the 5-HT concentration in the low-dose group (*P* = 0.031) and the high-dose group (*P* = 0.012) was significantly increased; Compared with the model group, the 5-HT concentration in the diazepam group (*P* = 0.002), the medium-dose group (*P* = 0.000), and the high-dose group (*P* = 0.000) was extremely significantly increased, and the 5-HT concentration of the low-dose (*P* = 0.193) group was. not significantly different. From the perspective of the concentration of GABA in the brain in mice, compared with the control group, the GABA concentration in the model group (*P* = 0.000), the diazepam group (*P* = 0.000) and the low-dose group (*P* = 0.000) was extremely significantly reduced, while there was no significant difference in the medium-dose group (*P* = 0.434) and the high-dose group (*P* = 0.114). Compared with the model group, except the low-dose group (*P* = 0.614), all the other groups showed significant differences (*P* < 0.01), which showed that the concentration of GABA in the brain increased.

### Compound Anshen essential oil component analysis

**Fig. 5:**
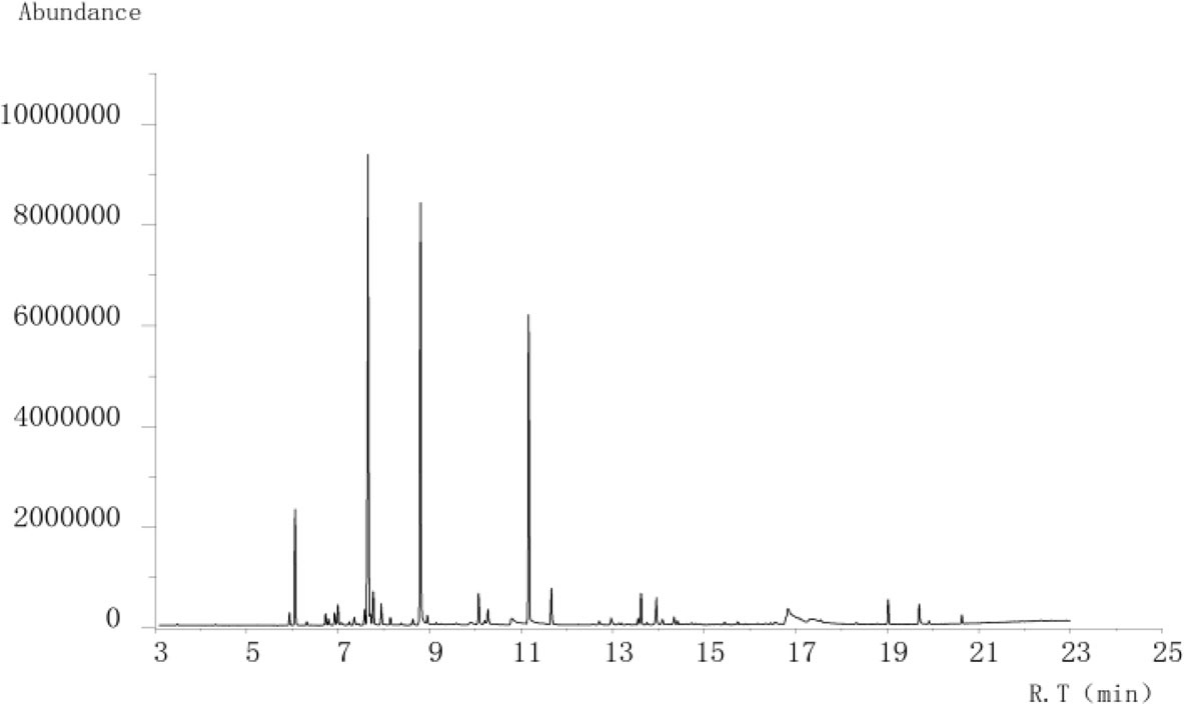
GC-MS analysis of the TIC component of Compound Anshen essential oil

The main chemical composition and relative percentage of Compound Anshen essential oil analyzed by GC-MS were shown in Table 1,GC-MS analysis of the TIC component of Compound Anshen essential oil is shown in Fig. 5.A total of 30 chemical components were identified, accounting for 93.92% of total volatile oil, with the highest content of D-limonene (24.07%), Linalool (21.98%), Linalyl acetate (15.37%), α-Pinene (5.39%), α-Santalol (4.8%), etc.

**Table 1:** Main chemical composition and percentage of Compound Anshen essential oil

No.	Retention Time (min)	Compound	Chemical formula	molecular weight	Relative Amount (%)
1	5.943	α-Thujene	C_10_H_16_	136	0.63
2	6.064	α-Pinene	C_10_H_16_	136	5.39
3	6.482	Sabinene	C_10_H_16_	136	0.28
4	6.733	β-Phellandrene	C_10_H_16_	136	0.58
5	6.796	β-Pinene	C_10_H_16_	136	0.4
6	6.927	3-Octanone	C_8_H_16_O	128	0.66
7	6.998	β-Myrcene	C_10_H_16_	136	1.04
8	7.079	Sarcosine	C_3_H_7_NO_2_	89	0.25
9	7.358	Hexyl acetate	C_4_H_8_O_2_	144	0.51
10	7.585	Cymene	C_10_H_14_	134	0.79
11	7.657	D-Limonene	C_10_H_16_	136	24.07
12	7.771	trans-β-Ocimene	C_10_H_16_	136	1.81
13	7.948	β-Ocimene	C_10_H_16_	136	1.17
14	8.148	γ-Terpinene	C_10_H_16_	136	0.41
15	8.803	Linalool	C_10_H_18_O	154	21.98
16	10.076	4-Terpineol	C_10_H_18_O	154	1.73
17	8.953	1-Octen-3-yl acetate	C_10_H_18_O_2_	170	0.63
18	10.076	Terpinen-4-ol	C_10_H_18_O	154	1.73
19	10.206	Cyclobutanol	C_4_H_8_O	72	0.26
20	10.28	α-Terpineol	C_10_H_18_O	154	0.95
21	10.812	Citronellol	C_10_H_20_O	156	1.42
22	11.171	Linalyl acetate	C_12_H_20_O_2_	196	15.37
23	11.666	lavandulyl acetate	C_12_H_20_O_2_	196	1.8
24	12.971	Geranyl acetate	C_12_H_20_O_2_	196	0.51
25	13.624	Caryophyllene	C_15_H_24_	204	1.71
26	13.958	β-Famesene	C_15_H_24_	204	1.57
27	14.094	β-santalene	C_15_H_24_	204	0.38
28	16.839	α-Santalol	C_15_H_24_O	220	4.8
28	19.023	Nonadecane	C_19_H_40_	268	1.21
29	19.702	Dibutyl phthalate	C_16_H_22_O_4_	278	1.21
30	20.636	Eicosane	C_20_H_42_	282	0.4

## Discussion

In order to study the sedative and hypnotic effects of Compound Anshen essential oil inhalation in the treatment of insomnia, animals need to establish a PCPA insomnia model. The control group and the PCPA model group did not implement insomnia treatment intervention, and other groups performed drug therapy intervention. Comparing and analyzing the experimental parameters of other groups and model groups, in order to explore the pharmacodynamics of Compound Anshen essential oil inhalation for insomnia. By comparing and analyzing the pharmacodynamics of Compound Anshen essential oil and diazepam, the difference between the sedative and hypnotic effects of Compound Anshen essential oil and diazepam was explored. Therefore, the sedative and hypnotic effects of Compound Anshen essential oil on animals were studied experimentally and compared with the diazepam group. In this experiment, the sedative and hypnotic effects of inhaling Compound Anshen essential oil were studied. Compared with the model group, mice in the low-dose group, the medium-dose group, the high-dose group and the diazepam group had glossy hair color, normal circadian rhythm and significantly increased body mass, among which the dose group had the best performance. To study the effects of inhaling Compound Anshen essential oil on spontaneous activity and pentobarbital-induced latency of sleeping time and duration of sleeping time in mice, the results showed that Compound Anshen essential oil could significantly reduce the autonomic activity. In general, decreased autonomic activity in mice indicates a sedative effect of the drug, and this change in behavior is thought to reflect a decreased excitability of the central nervous system. The inhibitory effect on autonomic activity indicates that Compound Anshen essential oil has a sedative and inhibitory effect on excitability. At the same time, Compound Anshen essential oil has synergistic effect with pentobarbital, reduce latency of sleeping time and prolong duration of sleeping time show the hypnotic effect of Compound Anshen essential oil.

Dysfunction of 5-HT, GABA and other neurotransmitters is closely related to insomnia. It has been reported that therapeutic drugs induce sedation and hypnosis by regulating neurotransmission, such as the 5-HTergic system or GABAergic system in the central nervous system [32]. Therefore, the analysis of 5-HT and GABA levels in the brain is of great significance for the detection of drugs for the treatment of insomnia [7]. Serotonin (5-HT) plays a key role in sleep-wake regulation [33]. Serotonin is thought to be a sleep neurotransmitter that produces sleep by inhibiting the midbrain’s reticular activating system or the norepinephrine component of the blue spot [34]. Conversely, PCPA, a tryptophan hydroxylase inhibitor, consumes 5-HT, leading to insomnia. After 24–36 h, PCPA had a strong inhibitory effect on 5-HT synthesis [35]. GABA is a major inhibitory neurotransmitter in the central neurosystem (CNS). GABA receptor system plays a major inhibitory role in the brain and plays a crucial role in regulating the overall balance between neuron excitation and inhibition [36]. Neurons in the front of the hypothalamus release GABA, which inhibits areas of the hypothalamus and brainstem that promote wakefulness [37]. Dysfunction or deficits in the GABAergic system are associated with epilepsy, pain and anxiety [38]. The GABAergic neurotransmitter plays a key role in sleep regulation. The BZD binding site on GABAA receptor is the target of most sedative and hypnotic drugs [39]. Barbiturates, such as pentobarbital, act on ion group complexes of GABA receptors, which are favorable for GABA binding. Benzodiazepines, such as diazepam, increase the affinity of GABA to its receptors, thereby increasing the duration of pentobarbital induced sleep [40]. It has been suggested that essential oil components play a sleep promoting role by regulating the 5-HTergic system or GABAergic system [41]. Therefore, the effect of Compound Anshen essential oil on the levels of 5-HT and GABA in the brain is of great significance for evaluating the pharmacodynamics of Compound Anshen essential oil for the treatment of insomnia. In addition to the above reasons, this experiment uses the PCPA insomnia model, because PCPA is a tryptophan hydroxylase inhibitor, consumes 5-HT, leading to insomnia, detection of brain 5-HT levels can reflect the successful establishment of insomnia model. In this study, the levels of 5-HT and GABA in the brain were measured by enzyme-linked immunosorbent assay. The results showed that the low-dose, middle-dose and high-dose groups of the Compound Anshen oil showed different degrees of 5-HT up-regulation and GABA up-regulation in a dose-dependent manner. The levels of 5-HT and GABA in the middle-dose and high-dose groups were similar to those in the control group and higher than those in the diazepam group. Therefore, the inhalation administration of the Compound Anshen essential oil can significantly increase the levels of 5-HT and GABA in the brain. From the experiment of weight change and the analysis of brain neurotransmitter level, the effect of Compound Anshen essential oil inhalation on insomnia is better than that of diazepam. It can be seen that inhalation of Compound Anshen essential oil may be a safe and effective treatment for or adjuvant treatment of insomnia, which may reduce the excessive use of prescription drugs such as diazepam and reduce the risk of short-term or long-term effects on sleep health. Its deeper sedative and hypnotic mechanism needs further study.

On this basis, the composition of Compound Anshen essential oil was analyzed by gas chromatography-mass spectrometry (GC-MS). A total of 30 chemical constituents were identified, accounting for 93.39% of total volatile oil. The main components of Compound Anshen essential oil are esters, alcohols, alkenes, alkyls and other compounds, with the highest content of D-limonene (24.07%), Linalool (21.98%), Linalyl acetate (15.37%), a-Pinene (5.39%) and α-Santalol (4.8%).D-limonene [42], Linalool [43], Linalyl acetate [44] and α-Santalol [45] have been reported to have central nervous sedative effect. In addition, D-limonene has anti-anxiety and soothing effects [46], Linalool has anti-anxiety and depression-like effects [47] and α-Pinene has anti-anxiety [48] and stress-relieving effects [49]. It is speculated that the sedative and hypnotic effect of Compound Anshen essential oil is related to its main chemical composition and activity. Based on the network pharmacology, the active components, targets and pathways of the Compound Anshen essential oil were predicted. The results of the network pharmacology analysis were published [50]. Among the volatile chemical constituents of the compound anshen essential oil traced by GC-MS, the active ingredients related to insomnia were screened and the relevant targets and pathways were predicted. The study found that Dibutyl phthalate, Caryophyllene, Geranyl acetate, Linalool, α-Terpineol, and Terpinen-4-ol played a key role in the treatment of insomnia in the Compound Anshen essential oil. The chemical components with the highest content tracked by GC-MS were D-Limonene, Linalyl acetate, α-Pinene, which were also correlated with treatment of insomnia. It is predicted that the compound anshen essential oil mainly exerts pharmacodynamic effects through related target proteins such as ESR1, GABRA1, GABRA2, GABRA3, GABRA4, GABRA5, NR1H4, CHRM1, SLC6A2, SLC6A3, SLC6A4, CYP3A4, DRD1, DRD2, OPRD1, OPRM1, HCRTR1, HTR2A,etc. It is mainly related to Calcium signaling pathway, Neuroactive ligand-receptor interaction, Cholinergic synapse, GABAergic synapse and other pathways. Based on the network pharmacology method, the active ingredients of the Compound Anshen essential oil have sedative, hypnotic, anti-anxiety and anti-depression effects, and the correlation between the active ingredients and the target and pathway. To some extent, the reliability of the study on the sedative and hypnotic effects of the Compound Anshen essential oil and the reliability of the GC-MS analysis of the chemical components were confirmed.

## Conclusion

The study found that the inhalation of Compound Anshen essential oil has sedative and hypnotic effect, which can significantly reduce autonomic activities, shorten latency of sleeping time and prolong duration of sleeping time, increase 5-HT and GABA content in the brain. The main chemical components of Compound Anshen essential oil contain many kinds of chemical components which have the sedative and hypnotic effect on the nerve center, anti-anxiety effect and anti-depression effect.

## Data Availability

The data sets used and/or analyzed during the current study available from the corresponding author on reasonable request.

## Abbreviation

CEO: Compound Anshen essential oil
